# Genome-wide identification and expression analysis of the *SAUR* gene family in foxtail millet (*Setaria italica* L.)

**DOI:** 10.1186/s12870-023-04055-8

**Published:** 2023-01-14

**Authors:** Xiaoqian Ma, Shutao Dai, Na Qin, Cancan Zhu, Jiafan Qin, Junxia Li

**Affiliations:** 1 College of Agriculture, Henan University of Science and Technology, Luoyang, 471000 Henan People’s Republic of China; 2 Henan Academy of Agriculture Sciences, Cereal Crops Institute, Zhengzhou, 450002 Henan People’s Republic of China; 3 Luoyang Academy of Agriculture and Forestry Sciences, Sweet Potato and Millet Institute, , Luoyang, 471023 Henan People’s Republic of China

**Keywords:** *Setaria italic*, *SAUR* gene family, Genome-wide identified, Expression profile, Abiotic stress

## Abstract

**Background:**

Auxin performs important functions in plant growth and development processes, as well as abiotic stress. *Small auxin-up RNA* (*SAUR*) is the largest gene family of auxin-responsive factors. However, the knowledge of the *SAUR* gene family in foxtail millet is largely obscure.

**Results:**

In the current study, 72 *SiSAUR* genes were identified and renamed according to their chromosomal distribution in the foxtail millet genome. These *SiSAUR* genes were unevenly distributed on nine chromosomes and were classified into three groups through phylogenetic tree analysis. Most of the *SiSAUR* members from the same group showed similar gene structure and motif composition characteristics. Analysis of *cis*-acting elements showed that many hormone and stress response elements were identified in the promoter region of *SiSAURs.* Gene replication analysis revealed that many *SiSAUR* genes were derived from gene duplication events. We also found that the expression of 10 *SiSAURs* was induced by abiotic stress and exogenous hormones, which indicated that *SiSAUR* genes may participated in complex physiological processes.

**Conclusions:**

Overall, these results will be valuable for further studies on the biological role of *SAUR* genes in foxtail development and response to stress conditions and may shed light on the improvement of the genetic breeding of foxtail millet.

**Supplementary Information:**

The online version contains supplementary material available at 10.1186/s12870-023-04055-8.

## Background

The phytohormone auxin is widely distributed in plants and can control cell elongation, division, expansion, differentiation, and so on, thus affecting various aspects of plant growth and development [1]. The early auxin-responsive genes were mainly composed of *Gretchen Hagen* 3 (*GH3*), *Auxin/Indoleacetic acid* (*Aux/IAA*) and *Small Auxin-Up RNA* (*SAUR*) gene families, among which *SAUR* was closely related to the early auxin-responsive genes. *GH3* plays an important role in both auxin and light signaling pathways, and also plays different roles in defense response [2]. *Aux/IAA* is a transcription inhibitor that provides a pathway for auxin signal transduction, and encoding proteins have also been proven to play a very important role in the auxin signal transduction pathway [3].

*SAUR* genes are extremely abundant in plants. The first *SAUR* gene was found in the hypocotyl of soybean [4]. Over the past few decades, members of the *SAUR* gene family have been identified in many plants, such as mung bean [5], tomato [6], Arabidopsis [7–9], apple [10, 11], maize [12, 13], rice [14], sorghum [15], potato [16], cotton [17, 18], and poplar [19]. Interestingly, most *SAUR* genes have only one exon and exist in clusters. *SAUR* genes expression can be induced rapidly by exogenous auxin within 2 to 5 min. Gene structure analysis showed that a conserved downstream element (DST) exists in the 3’-untranslated region of *SAUR*, which makes the encoded mRNA extremely unstable and can be degraded in a few minutes [3, 14, 16, 20]. However, the biological function of *SAURs* largely remains obscure.

Lots of *SAUR* genes have been shown to play an important role in plant growth, development, and stress response in Arabidopsis thaliana. Some *AtSAUR* genes are involved in auxin mediated cell expansion with their unique expression pattern. For example, Arabidopsis *AtSAUR36* [21], *AtSAUR41* [22], *AtSAUR19* [23], and *AtSAUR63* [24], positively regulate cell expansion and promote hypocotyl growth with high expression in hypocotyls, cotyledons, petioles, and flowers. Recent studies have shown that *AtSAUR41* is inducible by abscisic acid to regulate salt tolerance [22, 25]. Although the function of the *SAUR* gene family has been extensively reported in Arabidopsis thaliana, it is rarely reported in monocotyledonous rice. There are 58 *SAUR* gene members in rice [14], but only three members have been cloned: *OsSAUR39*, *OsSAUR45*, and *OsSAUR33*. Compared with the wild type, the transgenic plants overexpressing *OsSAUR39* had lower auxin content, reduced auxin polar transport, and showed a significant decrease in tiller and panicle number [26]. *OsSAUR45* affects plant growth by inhibiting the expression of *OsYUCCA* and *OsPIN* genes and affecting auxin synthesis and transport [27]. Disruption of *OsSAUR33* significantly reduced seed vigor and germination rate, but increased soluble sugar content in early germination and mature seeds [28]. However, a comprehensive investigation of *SAUR* family genes has not been conducted and their special role in auxin signaling is still unknown in foxtail millet.

Foxtail millet (*Setaria italic* L.) is an important minor crop due to its strong tolerance to drought and barren stress. For its small genome (~ 515 Mb) and short life cycle, foxtail millet has gradually developed into an ideal C4 model plant. Although genome sequencing has been completed in 2013 [29], the functional genomics of foxtail millet is still developing slowly. What we have known and understood in *SAUR* gene family is too little to comprehensively understand the genetic basis of *SAUR* genes. Also, the *SAUR* gene family has not been systematically identified and analyzed in foxtail millet. To better understand the function and evolution of the *SAURs* in plants, we analyzed the *SAUR* gene family in foxtail millet. A total of 72 *SiSAUR* genes were identified here and they were divided into three groups. In addition, comprehensive analysis, including gene structure, conserved motifs, *cis*-acting elements, gene replication, synteny analyses, expression analysis, and subcellular localization of these genes were all performed.

## Results

### Identification, characterization, and phylogenetic analysis of SAUR genes in foxtail millet

In our study, the HMM model of *SAUR* conserved domain was used to verify all possible *SAUR* members in the *S.italica* genome. The identified members of the *SiSAUR* gene family were renamed as *SiSAUR1* to *SiSAUR72* (Table S1)*.* Among the 72 SiSAUR proteins, SiSAUR26 was the smallest with 75 amino acids (aa), while the largest was SiSAUR13 with 373 aa. The molecular masses and isoelectronic point (pI) of the proteins ranged from 8.21 kDa to 39.49 kDa and from 4.9 (SiSAUR4) to 11.61 (SiSAUR13), with a mean of 8.27, respectively. Seventeen SiSAURs had a pI of less than 7, indicating that they were acidic proteins, while the pI of the rest of the proteins were all higher than 7, indicating that they were basic proteins. The instability index of SiSAUR proteins ranges from 26.57 (SiSAUR3) to 80.62 (SiSAUR10). Almost 90% (65/72) of the SiSAUR proteins had an instability index of more than 40, suggesting that SiSAUR proteins were extremely unstable, which was consistent with the report in [3, 14, 16]. The aliphatic index of SiSAUR proteins ranges from 53.19 (SiSAUR32) to 104.15 (SiSAUR4), with a mean of 78.55. Based on the average grand of hydropathicity, SiSAUR10, SiSAUR32, SiSAUR43, SiSAUR70, and SiSAUR71 were classified as hydrophilic proteins, while others belong to amphiphilic proteins (Table S1).

The prediction of the secondary structure of these SAUR proteins in millet showed that α-helix, extended strand, β-turn, and random curl were found in each member of the family (Table S2). Among them, α-helix and random curl, accounting for 20% ~ 60% of the total secondary structure, were the major constituent elements of the secondary structure of the gene family that favors the formation of the conformation of a particular protein structure. Extended strand accounted for 5% ~ 27%, β-turn accounted for the least, only at around 6%. Subcellular localization results suggested that 37 SiSAUR proteins were predicted to be in the mitochondria, 21 were predicted to be in the cytoplasm, and 14 were predicted to be in the nucleus. All the 72 *SiSAUR* family members obtained the conservative domain auxin-inducible (Table S2).

**Fig. 1:**
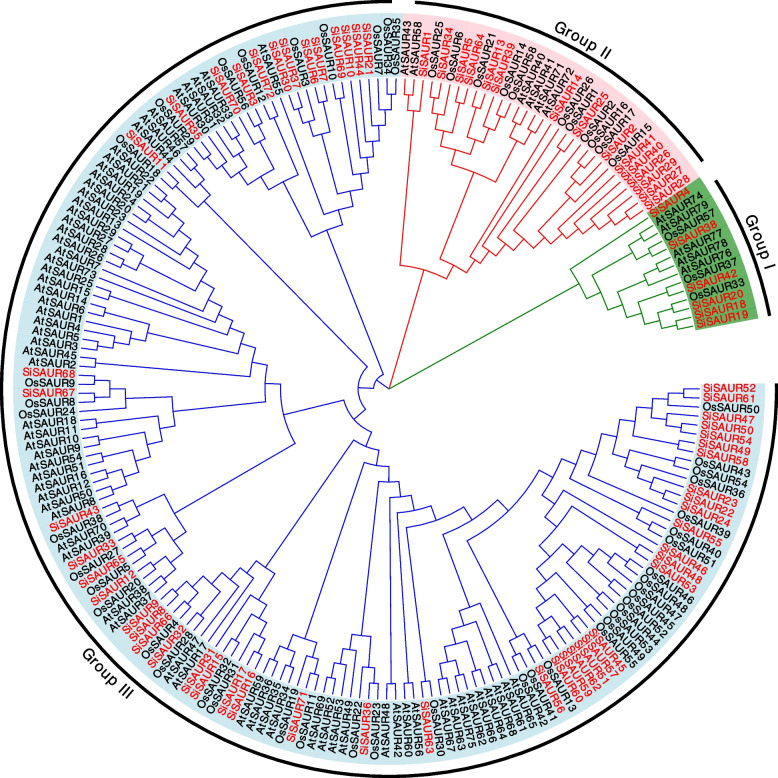
Phylogenetic tree of *SAUR* proteins in *S.italica*, *Arabidopsis* and rice. The phylogenetic tree was derived using MEGA11 software by neighbor-joining method with 1000 bootstrap replicates. Species abbreviations are listed as follows. At: *A. thaliana*; Os: *O. sativa*; Si: *S. italica*

**Fig. 2:**
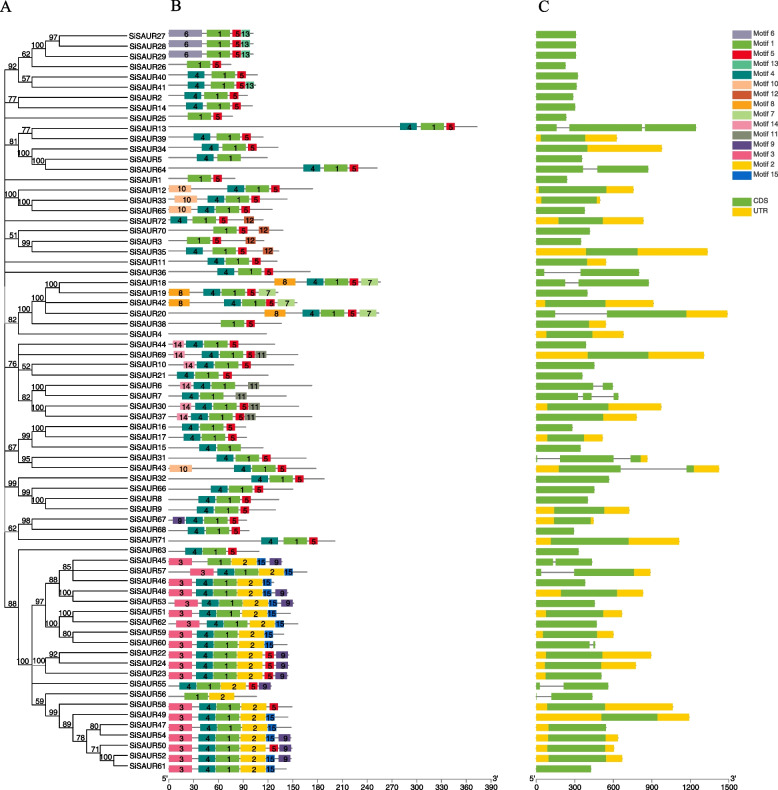
Phylogenetic relationship, architecture of conserved motifs and exon–intron structure in *SAUR* genes in foxtail millet. **A** The phylogenetic tree was constructed using MEGA11 software by neighbor-joining method with 1000 bootstrap replicates. **B** Distribution of conserved motifs in *SiSAUR* gene family. The number of motif 1–15 are displayed in different colored boxes. **C** The gene structure of *SiSAUR* genes. Untranslated region, exons and introns are displayed by colored boxes and gray lines, respectively. The length of protein was indicated using the scale at the bottom

To further investigate the phylogenetic relationship of SAUR proteins, phylogenetic tree was constructed using the protein sequences of the 72 identified *SiSAURs*, 79*AtSAURs* [3], and 58 *OsSAURs* [14] (Fig. 1, Table S1 and S3). The phylogenetic tree was divided into three main phylogenetic clades, namely Group I, Group II, and Group III. Among these members, the Group III branch was the largest group containing 163 members, Group II contained 32 members, while the Group I branch contained only 14 members (Fig. 1 and Fig. 2A).

### Conserved motifs, gene structures and cis-acting elements analysis of SiSAUR genes

The conserved motifs and distribution of exon–intron structures were analyzed to explore the structural diversity of *SiSAUR* genes (Fig. 2). A total of 15 conserved motifs (motif 1 to motif 15) were identified (Fig. 2B, Table S4). Except for *SiSAUR4*, all of the other *SiSAUR* genes contained a motif 1. Interestingly, *SiSAUR4* did not contain any motif. Motif 4 and motif 5 were widely distributed in the *SiSAUR* family. Interestingly, the motif distribution was different in the three groups of the *SiSAUR* gene family. Both groups contain motifs 1, 4, and 5, motifs 7 and 8 appear in Group I, motifs 6 and 13 appear in Group II, while motifs 2–3, 9–12, and 14–15 appear in Group III. Although there were differences in motif types between groups, members of the same group such as *SiSAUR28* and *SiSAUR29*, *SiSAUR2* and *SiSAUR14*, *SiSAUR46* and *SiSAUR57* tend to exhibit similar motif patterns (Fig. 2A, B), indicating functional similarity between them. The comparison of the number of the exon–intron structures revealed that the 72 *SiSAUR* genes had different numbers of exons, varying from 1 to 3 (Fig. 2C, Table S1). Most of them (59, ~ 81.9%) contained no intron; 11 *SiSAUR* genes contained one intron; only *SiSAUR7* and *SiSAUR13* contained two introns (Fig. 2C). Exon–intron structural analysis showed that most members of the *SiSAUR* gene family have no intron structures, which was consistent with the results in rice [14] and other studies [8, 9].

**Fig. 3:**
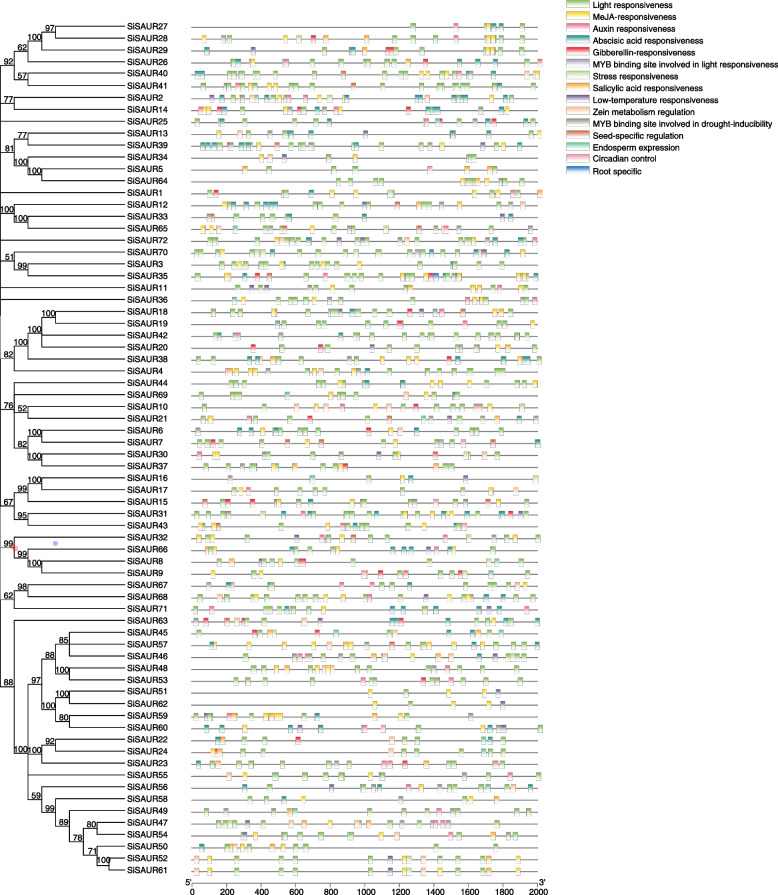
*Cis*-elements prediction in the 2000 bp region upstream from the start codon of *SiSAURs*

**Fig. 4:**
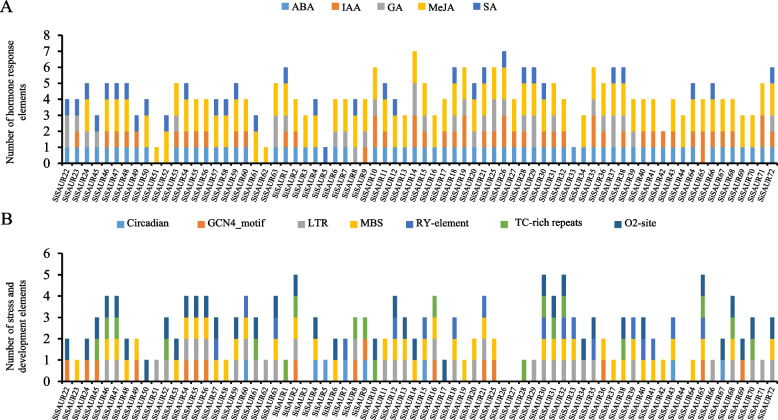
The number of plant hormone and induced stress *cis*-acting elements in the promoter region of *SiSAURs*. **A** The sum of hormone response *cis*-acting elements in each member of *SiSAURs*. **B** The sum of *cis*-acting elements of stress and development in each member of *SiSAURs*

The *cis*-acting elements in the promoter regions (the upstream 2000 bp from the initiation codon) of 72 *SiSAUR* genes were further investigated. A total of 46 *cis*-regulatory elements were identified (Fig. 3, Table S5), which were mainly divided into three categories: hormone response elements, stress response elements, and plant growth and development elements. There are five hormone-responsive elements in the *SiSAUR* gene family of the foxtail millet, which cover most plant hormones, including abscisic acid (ABA), auxin (IAA), gibberellin (GA), methyl jasmonate (MeJA), and salicylic acid (SA) (Fig. 4A). The abscisic acid responsiveness elements (ABRE elements, 64 members), light responsiveness elements (G-box element, 69 members) and MeJA-responsiveness elements (CGTCA-motif and TGACG-motif elements, 65 and 63 members) in the promoter region were identified in almost all the *SiSAUR* genes. Most of the *SiSAUR* gene promoter region contained three to four hormone response elements. *SiSAUR1*, *SiSAUR18*, *SiSAUR21*, *SiSAUR26*, *SiSAUR28*, *SiSAUR38*, and *SiSAUR72* contained all the five hormone-responsive elements. The MeJA response element had the largest number among the five hormone-responsive elements, followed by the ABA response element. The auxin response element was ranked third. Literatures reported that MeJA and ABA respond to stress [30], so the stress response and plant growth and development related elements were further studied.

As shown in Fig. 4B, *SiSAUR2*, *SiSAUR30*, *SiSAUR32*, and *SiSAUR65* contained the largest number of elements, including endosperm expression (GCN4_motif), low temperature response (LRT), MYB binding site (MBS), seed specific regulation (RY-element), stress responsiveness (TC-rich repeats), and zein metabolism regulation elements (O_2_-site). *SiSAUR3*, *SiSAUR26*, *SiSAUR27*, and *SiSAUR44* did not contain any stress elements or plant growth and development elements. Also, the number of genes containing MYB binding site (MBS) elements was the highest, followed by zein metabolism regulation (O_2_-site) and low temperature response (LRT) elements. These results indicated that the *SiSAUR* genes may be involved in stress response. In addition, some *cis*-acting elements may regulate the expression of different tissues (seed, root, and endosperm) during development (Table S5). These results suggested that *SiSAUR* genes could not only participate in the process of plant growth and development but also respond to various abiotic stresses.

### Chromosome locations and gene duplication analysis

**Fig. 5:**
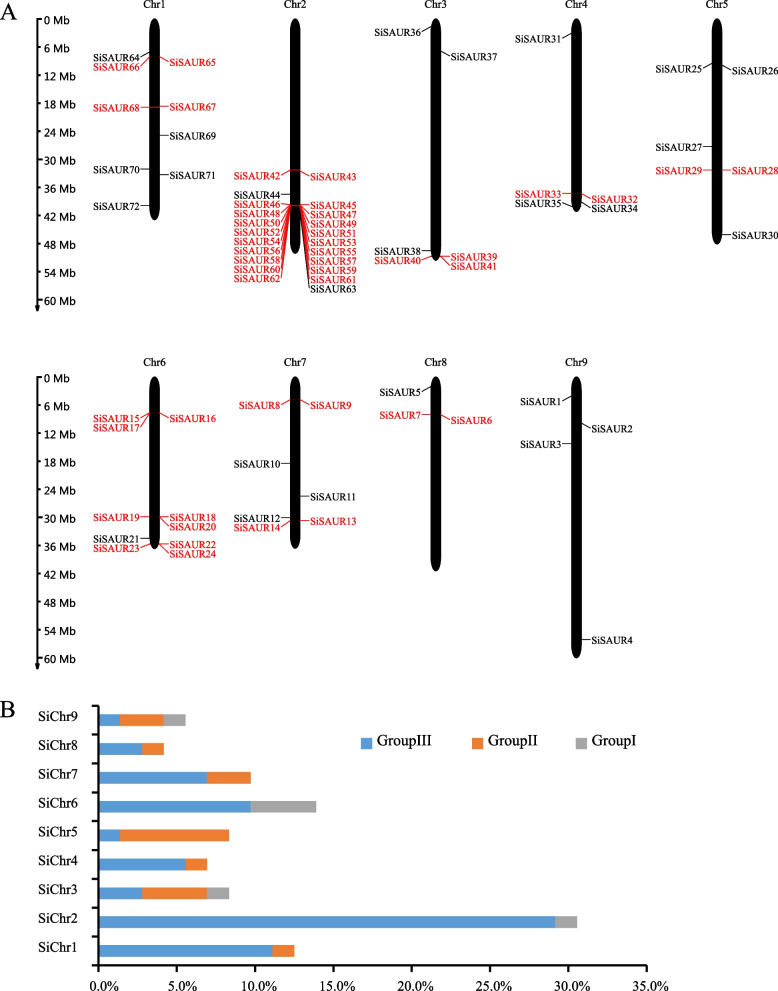
Chromosome distribution of *SiSAURs* in *S.italica*. **A** The 72 *SiSAUR* genes distributed on the nine foxtail millet chromosome. The tandem duplicated genes are colored and connected in red color and red lines. Vertical bars represent the chromosomes of *S.italica*. The chromosome number is indicated by the top of each chromosome. The scale on the left represents chromosome length. **B** The percentage of different group *SiSAUR* genes on nine chromosomes

The number and location of *SiSAUR* genes on the chromosomes were also investigated (Fig. 5A, Table S1). The physical positions of these *SiSAUR* genes on the chromosomes were visualized using the MapGene2Chrom website. The 72 *SiSAUR* genes are distributed on nine chromosomes unevenly. Chr2 contained the largest number of *SiSAUR* genes (22 genes, ~ 30.5%), followed by Chr6 (10, ~ 13.8%); Chr8 contained the fewest *SiSAUR* genes (3 genes, ~ 4.16%). Chr3 and Chr5 contained the same number of *SiSAUR* genes (six each, ~ 8.3%). Chr1, Chr7, Chr4, and Chr9 contained nine (~ 12.5%), seven (~ 9.7%), five (~ 6.9%), and four (~ 5.5%) *SiSAUR* genes, respectively. The *SiSAUR* genes in three groups also showed an uneven distribution. The genes in Group I were found on Chr2, Chr3, Chr6 and Chr9, whereas the genes in Group III were distributed across all nine chromosomes. There are more Group III genes on Chr2 (Fig. 5B). Also, some *SiSAUR* genes within the same group tend to cluster together on the chromosome. For example, *SiSAUR22* and *SiSAUR23*, and *SiSAUR13* and *SiSAUR14*, which belong to the Group III and Group II, respectively, are tightly linked on chromosomes 6 (*SiSAUR22/23*) and 7 (*SiSAUR13/14*) (Fig. 5).

**Fig. 6:**
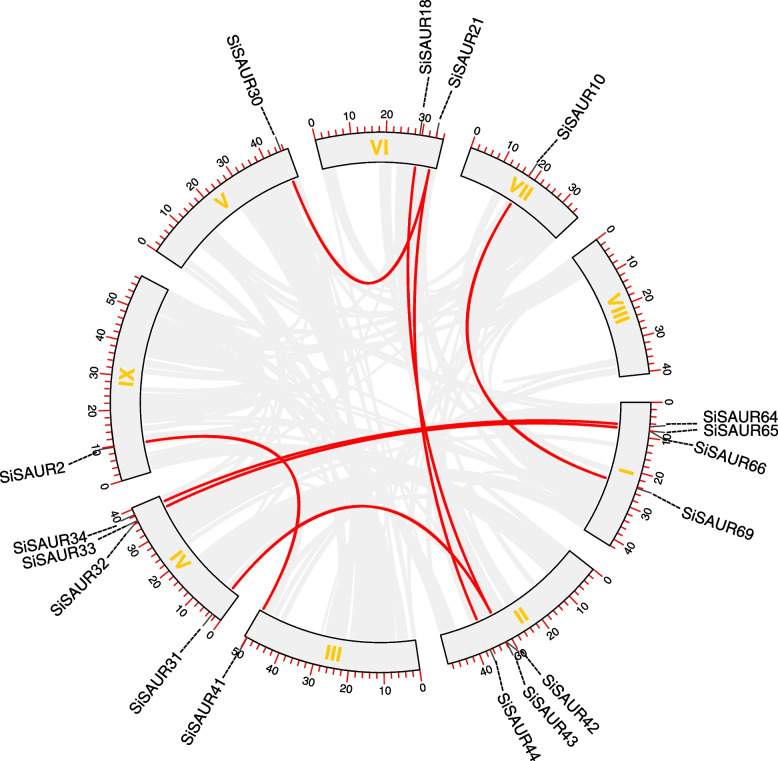
Schematic representations of the chromosomal distribution and segmental duplication relationships of *S. italica SAUR* genes. The red lines indicate duplicated *SAUR* gene pairs. The chromosome number is indicated on the inside of each chromosome

Gene duplication events, which generally include tandem repeats and segmental repeats, are always important for the expansion of gene families [31, 32]. Previous literature reported that the inclusion of two or more genes in a 200 kb region was defined as a tandem duplication event [33]. In this study, we found 33 tandem duplication events involving 46 *SiSAUR* genes on all the chromosomes except chromosome 9 (Fig. 5A). *SiSAUR16, SiSAUR19, SiSAUR23,* and *SiSAUR40* each involved in two tandem repeat events (*SiSAUR16* and *SiSAUR15*/*SiSAUR17*; *SiSAUR19* and *SiSAUR18*/*SiSAUR20*; *SiSAUR23* and *SiSAUR22*/*SiSAUR24*; *SiSAUR40* and *SiSAUR39*/*SiSAUR41*). Interestingly, 18 *SiSAUR* genes formed 17 tandem duplication events on chromosome 2. All the *SiSAUR* genes involving in the tandem repeat events tend to belong to the same subgroup (Table S1). In addition, nine pairs of segmentally duplicated genes were found in the *SiSAUR* family (Fig. 6, Table S6). The nine segmental duplications were unevenly distributed into eight linkage groups (LG). The distribution of *SiSAUR* genes was largest in LG I and LG IV (each 4). LG II and LG V, and LG VI had three and two *SiSAUR* genes, whereas LG III, LG VII, and LG IX had only one *SiSAUR* gene (Table S6). These findings showed that some *SiSAUR* genes might be due to some events in gene replication, which might have been the primary driving factor for *SiSAUR* gene development and evolution.

### Synteny analyses of SiSAUR genes

**Fig. 7:**
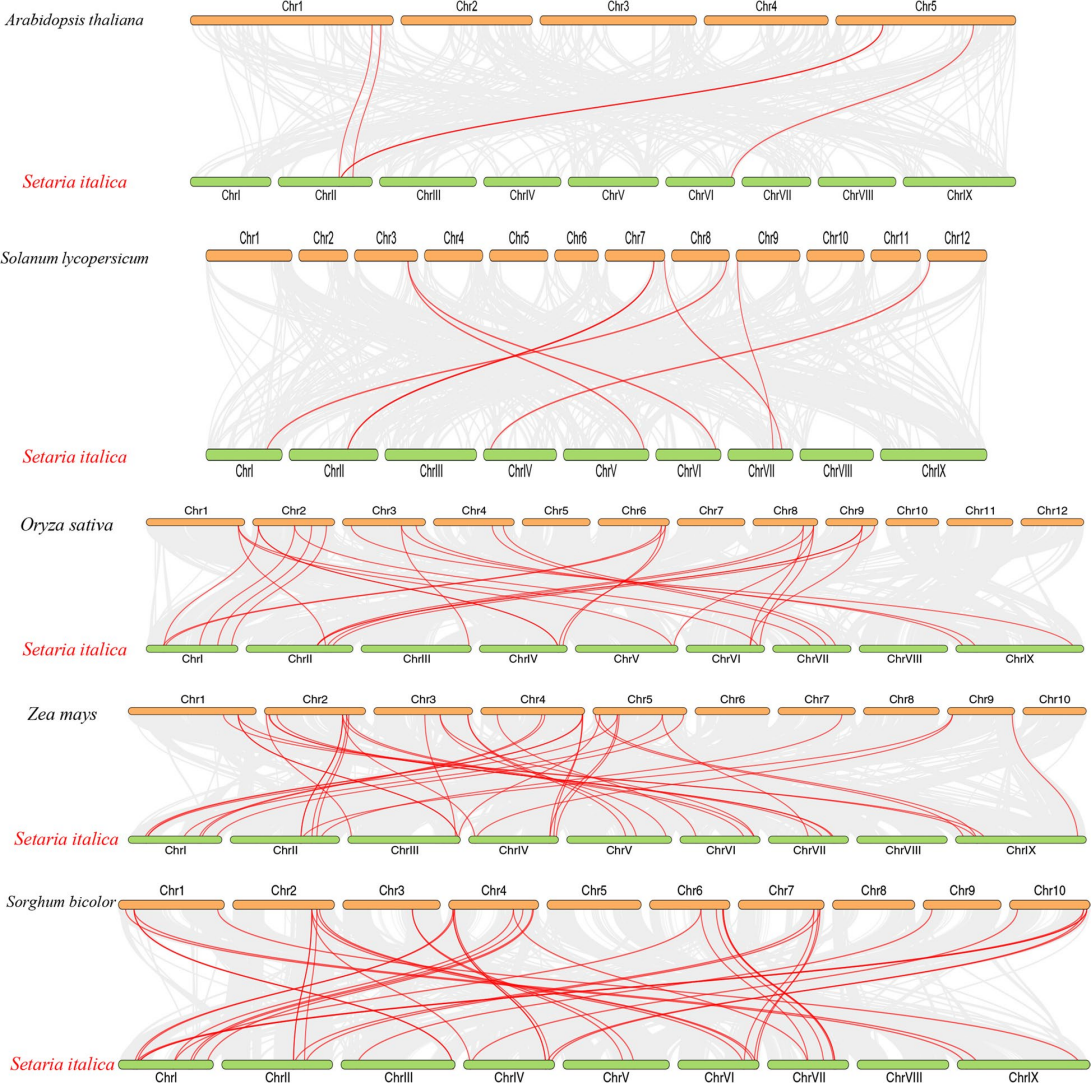
Synteny analyses of the *SAUR* genes between *S. italica* and five representative plant species. Gray lines on the background indicate the collinear blocks within *S. italica* and other plant genomes; the syntenic *S. italica SAUR* gene pairs were colored in red lines

To investigate syntenic relationship of *SiSAUR* genes, the collinearity analysis was conducted between *SiSAUR* genes and five other representative species (three monocotyledons: *Oryza sativa*, *Zea mays*, and *Sorghum bicolor*; two diocotyledons: *Arabidopsis thaliana* and *Solanum lycopersicum*) (Fig. 7, Table S7). We found that totals of 41 *SiSAUR* genes showed collinear relationships with *Arabidopsis* (4), *Solanum lycopersicum* (8), *Sorghum bicolor* (30), *Oryza sativa* (18), and *Zea mays* (35). The collinearity map revealed that *SiSAUR* genes showed the highest collinearity with maize, followed by *Sorghum bicolor*, rice, *Solanum lycopersicum* and *Arabidopsis*. Further analysis of these collinear genes revealed that some *SiSAUR* genes were found to exist in more than one collinear gene pair in five species, such as *SiSAUR42* with *AT1G72430.1/ Os09t0437100-00/ Zm00001eb100000_T001/ OQU89560/ Solyc07g042490.1.1*, which indicated that these collinear genes may have existed prior to the ancestral separation and divergence. What’s more, six *SiSAUR* genes (*SiSAUR11*, *14*, *21*, *31*, *43* and *71*) are present in both monocotyledons and diocotyledons (Table S7). Among these six genes, three genes (*SiSAUR21*, *31*, and *43*) were also included in the genes with segmental duplications (Fig. 5A, Table S6). And some *SiSAUR* genes showed collinearity only with maize (eight genes: *SiSAUR3*, *17*, *27*, *35*, *36*, *38*, *40*, and *63*) and *Sorghum bicolor* (five genes: *SiSAUR13*, *22*, *23*, *28*, and *37*) (C4 plant). In addition, the number of genes with collinearity to the monocots was much higher than that with collinearity to the dicots. The result that foxtail millet exhibited the highest collinearity with maize and *Sorghum bicolor*, suggesting that these C4 plants may have a closer genetic relationship.

### Organ expression pattern analysis of SiSAURs

**Fig. 8:**
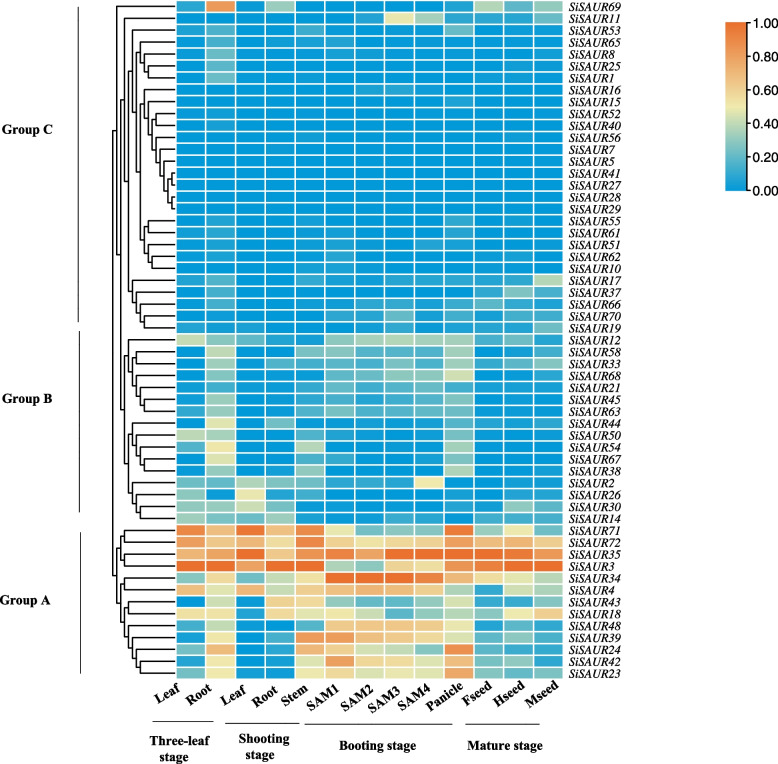
Heatmap of *SiSAUR* gene family at different stages in different tissues of foxtail millet Yugu1. Three-leaf stage: leaf, root. Shooting stage: leaf, root, stem. Booting stage: SAM1: SAM (0.5 cm), SAM2: SAM (1 cm), SAM3: SAM (1.5 cm), SAM4: SAM (2 cm), young panicle. Mature stage: Fseed: milking seed, Hseed: hard seed, Mseed: mature seed

To determine the expression of *SiSAURs* in various tissues of foxtail millet, the transcription data of *SiSAURs* in different tissues of Yugu1 were analyzed (Fig. 8). As shown in Fig. 8, the expression level of *SiSAURs* in different stages and tissues is mainly divided into three groups. The genes in group A were highly expressed in the whole development stage of foxtail millet. *SiSAUR71*, *SiSAUR72*, *SiSAUR35*, and *SiSAUR3* were highly expressed in root, stem, leaf, panicle, and seed, indicating that these four genes may play an important role during the growth and development of foxtail millet. *SiSAUR34* was specifically highly expressed in developing panicles, which may be related to panicle development. Group B genes have a relatively low expression level in the vegetative growth stage. Some group C gene expressions were not detected in any stage or tissue, such as *SiSAUR27*, *SiSAUR28*, and so on. The different expression patterns suggested that the functions of *SiSAURs* had been differentiated in long-term evolution.

### SiSAUR gene expression was induced by multiple abiotic stresses

**Fig. 9:**
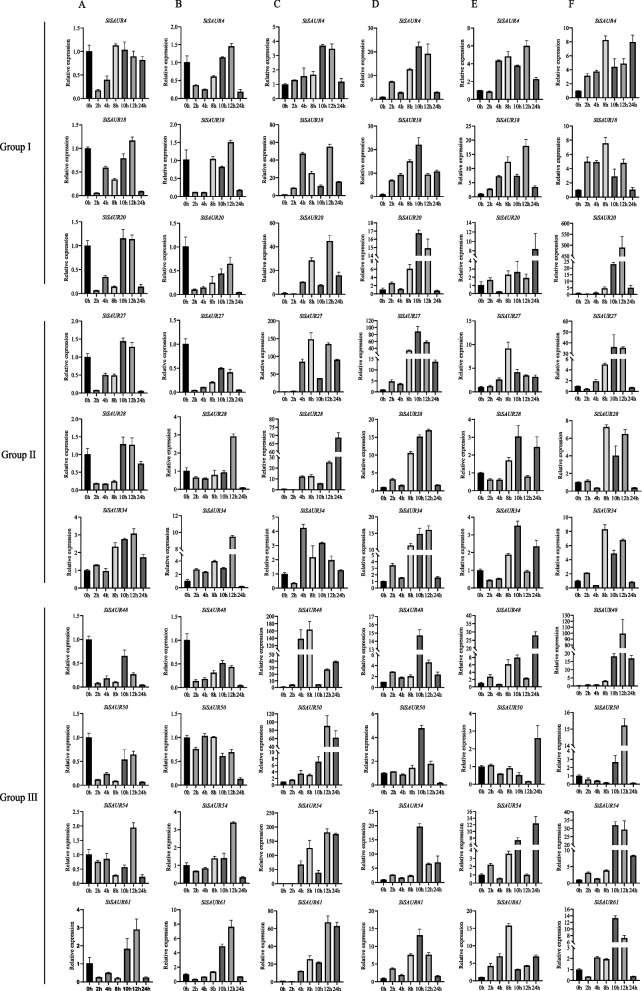
Expression profiles of *SiSAURs* under ABA (abscisic acid) **A**, GA (gibberellin) **B**, SA (salicylic acid) **C**, IAA (auxin) **D**, PEG (polyethylene glycol) **E**, NaCl **F** treatment. Yugu1 seedling shoots were sampled after 0, 2,4,8,10,12 and 24 h under stress conditions. The expression level of the *Siactin7* gene was used as the internal control.The values were the mean ± SE from three replication

According to the analysis of *cis*-elements in the promoter region of *SiSAURs*, *SiSAURs* might be involved in the abiotic stresses and hormone response processes. To further verify this hypothesis, the expression patterns of 10 *SiSAUR* genes belonging to Group I (*SiSAUR4*, *SiSAUR18*, *SiSAUR20*), Group II (*SiSAUR27*, *SiSAUR28*, *SiSAUR34*) and Group III (*SiSAUR48*, *SiSAUR50*, *SiSAUR54*, *SiSAUR61*), which were treated with 20% PEG-6000, 150 mM NaCl, ABA, SA, and GA, respectively, were detected by RT-qPCR (Fig. 9). These 10 *SiSAUR* genes had different responses to the various abiotic stresses and phytohormone treatments. They showed various expression patterns over time under different treatments. For example, after 2 h of ABA treatment, the expression of *SiSAUR4* and *SiSAUR28* genes was significantly inhibited, then recovered with the increase of treatment time. The expression of the *SiSAUR34* gene was significantly induced, and *SiSAUR18* and *SiSAUR27* showed a similar induced expression pattern, which was inhibited after 2 h of ABA treatment, then recovered rapidly, and after 24 h of treatment, the gene expression was inhibited again (Fig. 9A). Under GA treatment, the expression patterns of these 10 genes showed a trend of down-regulation first, then up-regulation and then down-regulation, similar to the expression pattern of *SiSAUR18* and *SiSAUR27* under ABA treatment (Fig. 9B). Under SA treatment, the expression level of *SiSAUR4* was significantly induced. *SiSAUR28*, *SiSAUR34*, *SiSAUR27*, *SiSAUR54*, and *SiSAUR61* showed different degrees of expression change, which was inhibited for 2 h after treatment, and then significantly up-regulated (Fig. 9C). When treated with 150 mM IAA, these 10 genes were all up-regulated, and the gene expression reached its highest level after 10 h of treatment (Fig. 9D). The obvious upregulation of the expression level under IAA treatment may be due to the fact that the promoter region of these *SAUR* genes is rich in a large number of auxin *cis*-acting elements (Fig. 3 and Fig. 4A). In PEG and salt stress conditions, these genes were induced to different degrees (Fig. 9E and F). The expression levels of *SiSAUR4* and *SiSAUR18* were significantly up-regulated under 150 mM NaCl and 20% PEG-6000 treatments, indicating that these two genes may be involved in drought stress. These results suggested that *SiSAUR* genes play various regulatory roles in abiotic stress and phytohormone treatment.

### Subcellular localization of SiSAUR proteins

**Fig. 10:**
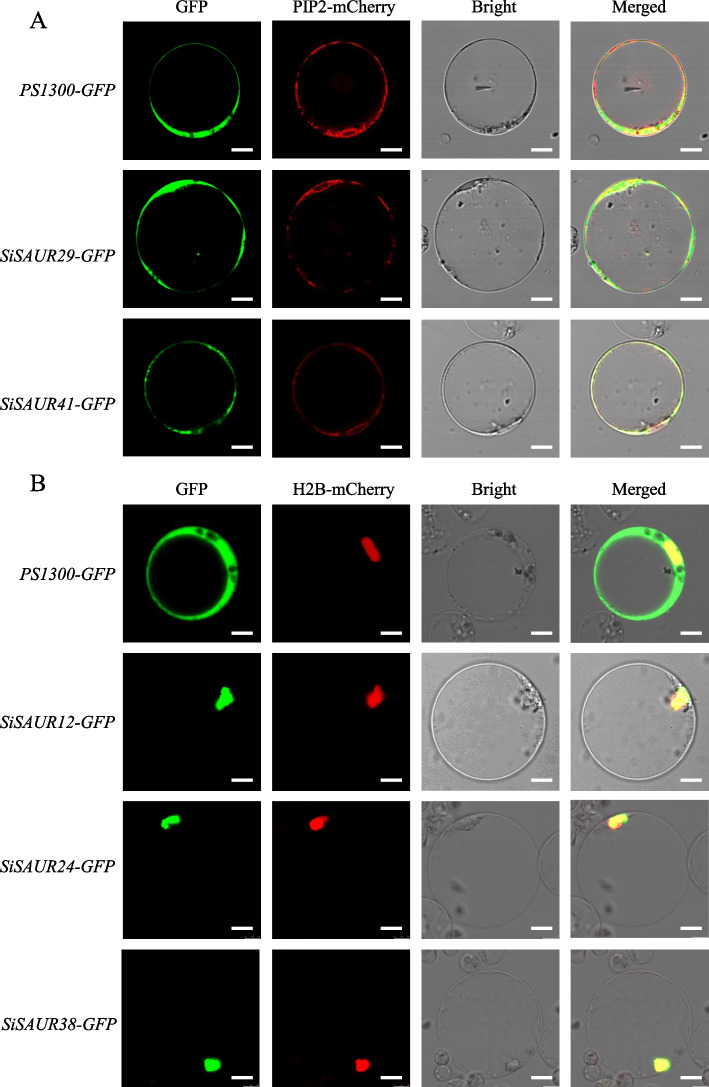
Subcellular localization of SAUR proteins. *ProSuper::GFP* (*PS1300*) vector was used as control. PIP2-mCherry: plasma membrane marker and H2B-mCherry: nuclear marker. Bar, 10 μm

According to the phylogenetic tree result, five genes, which came from different group (Group I: *SiSAUR38*; Group II: *SiSAUR29*, *SiSAUR41*; Group III: *SiSAUR12*, *SiSAUR24*), were picked to detect the location of these SiSAUR proteins (Fig. 10). The coding region with the stop codon removed was cloned into the PS1300 vector carrying the green fluorescent protein (GFP) gene, and then transformed into rice protoplast cell. The results showed that the GFP fluorescence signal of SiSAUR29-GFP was widely present in the cell membrane, nucleus, and cytoplasm, while SiSAUR41-GFP signal was found in the cell membrane, nuclear membrane, and cytoplasm (Fig. 10A). The GFP fluorescence signal of SiSAUR12, SiSAUR24 and SiSAUR38 were just existed in nucleus (Fig. 10B).

## Discussion

### SiSAUR gene structure and its evolutionary analysis

In this study, the *SiSAUR* gene family was thoroughly investigated, and over all 72 *SiSAUR* genes were discovered (Table S1). The SAUR protein has a length range from 75 to 373 amino acids, which may explain the sequence variability and complexity of SAUR. Among the 72 SAUR genes identified, 59 genes lacked intron, which also appeared in rice, sorghum, and other crops [14, 15, 17, 18]. Thirteen genes had introns, which might be involved in some biological processes like mRNA output and alternative splicing [34]. The imbalanced distribution of *SAUR* genes across each chromosome could be a sign that the genetic diversity has been there throughout the course of evolution. These *SAUR* genes were classified into three groups based on their evolutionary links with the known *Arabidopsis* and rice *SAUR* genes, indicating the indispensable role of these *SAUR* genes in the development and evolution of *S. italica* (Fig. 1, Table S1 and S3). According to the evolutionary tree, group III had a larger number of *S. italica* members (n = 51, 70.8%), similar to that in *Arabidopsis* (n = 68, 86.1%) and rice (n = 51, 70.8%), suggesting that these *SAUR* genes in group III may have undergone a more rapid expansion during the long process of evolution under the influence of monocotyledons.

Additionally, each group’s gene members not only share similar gene structure, motif structure, and protein length, but also have the same outcomes in predicting subcellular localization (Table S1 and S2). These findings imply that the *SAUR* gene family members, particularly those belonging to the same subfamily, may play more stable roles in foxtail millet growth. The similarities and variations in the gene structure, motif, and domain of *SiSAURs* may be linked to the extended evolutionary history as well as gene replication of foxtail millet. Segmented replication and tandem repetition are the main driving forces in the development of gene family extension and genomic evolution [31]. We discovered 33 events of tandem repeat comparising 46 *SiSAUR* genes (Fig. 5A) and nine segment duplication pairs (Fig. 6, Table S6). Therefore, *SiSAUR* gene tandem duplication might have a stronger impact on the *SAUR* family’s amplification and evolution in foxtail millet. As expected, all these genes were mainly within the same group, similar to *A.thaliana* [8, 9], and rice [14]. Collinear analysis also displayed that a majority of the *SAUR* homologous gene pairs existed in foxtail millet, *Zea mays* and *Sorghum bicolor* (Fig. 7, Table S7). Based on these findings, we hypothesized that whole-genome replication may be the key factor encouraging the proliferation of the *SAUR* genes.

### Expression patterns of SiSAURs under abiotic stresses

Here, qRT-PCR analysis was conducted to reveal the expression characteristics of *SiSAUR* genes under multiple phytohormone and abiotic treatments. Among these ten *SiSAUR* genes selected, the expression of *SiSAUR48* was obviously induced under various treatments (Fig. 9). Significant intron loss was clearly evident in most *SiSAUR* members (Table S1). These could result in family expansion and give rise to new roles and functions. In plants, genes having few or no introns are normally expressed at low levels. Compact-structured genes, on the other hand, may facilitate and ease the rapid expression of genes in response to abiotic stress [35]. For example, the expression of *SiSAUR4* increases rapidly under phytohormone, PEG, and NaCl stress treatments, and may be in response to these abiotic stresses (Fig. 9).

It is well known that the promoter regulates the temporal and spatial expression of a gene, and the *cis*-acting elements in the promoter region are crucial for gene function. Therefore, the *cis*-acting elements in the promoter region of *SiSAUR* genes were analyzed and large numbers of stress-responsive *cis*-acting elements, including hormone response elements, stress response elements, and plant growth and development elements, were found (Fig. 3, Table S5). ABA has been shown in studies to play a very crucial role in regulating stress responses in plants [36]. ABA response elements (ABREs) were identified in the *cis*-acting elements of the *SiSAUR* gene family. Also, qRT-PCR analysis revealed that many *SiSAURs* positively responded to stress in the short time of salt and drought treatments (Fig. 9E and F). Meanwhile, the qRT-PCR results of the *SiSAUR* family members indicated that they could be induced by various abiotic stresses, which were well consistent with previous reported results [37, 38]. These results suggest that the *SiSAUR* gene family may regulate the drought and salt stress responses, especially for genes *SiSAUR4*, *SiSAUR18*, *SiSAUR27*, *SiSAUR48*, and *SiSAUR54*, which need further experimental verification.

## Conclusions

A total of 72 *SAUR* genes have been discovered in the genome of foxtail millet. The characteristics, chromosomal distribution, phylogenetic relationships, conserved motifs, gene structure, *cis*-acting elements, gene replication, and synteny analyses of the 72 *SiSAUR* genes were characterized. These *SiSAUR* genes have been distributed unevenly across nine chromosomes and organized into three groups. The structure analysis of *SiSAUR* genes also revealed that the majority of them have no introns, suggesting that they are conserved. In addition, we discovered that both tandem and segment duplications are significant contributors to the expansion of the *SiSAUR* gene family, with tandem duplication possibly having a greater impact. In addition, the expression patterns of 10 *SiSAUR* genes were analyzed in response to different abiotic stresses, including PEG and NaCl stress, and different phytohormone treatments, including IAA, ABA, GA, and SA. The expression levels of these ten *SiSAUR* genes were all upregulated after IAA treatment, and these genes were sensitive to abiotic stresses. These results provide evidence of the relationship between *SiSAUR* genes and abiotic stresses, and they may be a valuable resource for traditional foxtail millet breeding.

## Methods

### Identification of the SiSAUR family genes

The complete genome of Foxtail millet was retrieved from the Ensemble Plants Genomes website (https://plants.ensembl.org/index.html). Foxtail millet *SAUR* sequences were downloaded through BLAST methods. First, was used to validate the candidate SAUR proteins and then we downloaded the hidden Markov model (HMM) file corresponding to the SAUR domain (*PF02519*) from the database of the Pfam protein family (http://pfam.xfam.org/). The SAUR protein sequences were obtained using HMMER3.0 with a cutoff of 0.01 (http://plants.ensembl.org/hmmer/index.html) from the foxtail millet genomic database. Finally, the NCBI Conserved Domain Database (https://www.ncbi.nlm.nih.gov/Structure/cdd/wrpsb.cgi) and SMART database (http://smart.embl-heidelberg.de/) were used to confirm whether the candidate protein sequences contained the *SAUR* core domain. Moreover, the basic characteristics of the trihelix proteins encoded by the *SAUR* genes of *S. italica* were determined using ExPasy (https://web.expasy.org/protparam/). SOPMA (https://npsa-prabi.ibcp.fr/cgi-bin/npsa_automat.pl?page=npsa_sopma.html) and Psort (https://www.genscript.com/psort.html) were used to predict the secondary structure and subcellular localization of SAUR proteins, respectively.

### Phylogenetics, gene structures and conserved motif analysis

The phylogenetic tree was generated using the Neighbor-Joining (NJ) method with 1000 bootstrap replications in MEGA11 [39]. The Evolview online tool was used to annotate and visualize the resulting tree [40]. Also, the predicted coding sequences were compared to their full-length sequences through the online program Gene Structure Display Server (http://gsds.gao-lab.org/index.php), which was used to determine the exon–intron structure of *SiSAUR* genes [41]. The Multiple EM for Motif Elicitation (https://meme-suite.org/index.html) online program was used to discover the conserved motif in the *SiSAUR* gene proteins [42]. These parameters were carried out to search for: the number of motifs is 20, while the motif width ranges from 6 to 50 residues. Finally, the outcomes of the gene structure as well as conserved motif analysis were visualized using TBtools software [43].

### Cis-regulatory element analysis of SiSAUR genes

The complete genome of foxtail millet was retrieved from the Ensemble Plants Genomes website (https://plants.ensembl.org/index.html). TBtools software was used to obtain the 2000 bp sequence upstream of the initiation codon of *SiSAUR* genes. Then PlantCARE software was then used to predict the 2000 bp upstream cis-acting elements in the *SiSAUR* genes (http://bioinformatics.psb.ugent.be/webtools/plantcare/html/). Finally, the results were visualized using TBtools software.

### Chromosomal distribution and gene duplication

The physical locations of *SiSAUR* genes were determined using the genome annotation files, which were downloaded from the Ensemble Plants Genomes website. The *SiSAUR* gene positions on the chromosome were visualized through MG2C software (http://mg2c.iask.in/mg2c_v2.1/). The TBtools software was used to scan the collinearity of *SiSAUR* genes and analyze gene-duplication events. TBtools was also used to analyze the collinear relationship between *SAUR* genes of foxtail millet, maize, rice, *Arabidopsis*, *Solanum lycopersicum*, and *Sorghum bicolor* by default parameters, and the collinear gene pairs between millet and maize, rice, *Arabidopsis*, *Solanum lycopersicum*, and *Sorghum bicolor* were obtained.

### Plant materials, growth conditions and treatments

In our study, the foxtail millet accession (Yugu 1) was used and obtained from Li Junxia of Henan Academy of Agricultural Sciences. The seeds of Yugu1 were soaked overnight and pregerminated on moistened filters in a plant growth chamber under the condition of 16 h light/8 h dark (28 °C/24 °C) with 65% humidity for 3 days. Then the seedlings were transferred into a hydroponic box with a modified Hoagland nutrient solution. When the third leaf was unfolded (approximately 4 weeks), the seedlings were treated with 20% PEG-6000, 150 mM NaCl, 150 mM IAA, 150 mM SA,150 mM ABA and 150 mM GA. Samples were taken at 0, 2, 4, 8, 10, 12 and 24 h, respectively. All of the samples were frozen immediately in liquid nitrogen and stored at -80 °C for further assays.

### RNA extraction and qRT-PCR analysis

Total RNA was extracted from shoots of each treatment using RNAiso Plus (Takara, Japan), and cDNA was generated using an M-MLV reverse transcriptase (Takara, Japan). qRT-PCR was performed on an ABI 7500 Real-time PCR system (Applied Bio-Systems). *SiActin7* was used as an internal reference. Each experiment was performed with five biological samples, and each sample was assayed with three technical replications. The relevant gene primers are listed in Table S8. The experimental data ware analyzed using the 2^−(△△CT)^ method [44].

### Vector construction and subcellular localization

The coding sequences without the termination codon of these five *SiSAUR* genes were cloned into the PS1300-GFP vector. The empty plasmid and fusion plasmids were co-transformed into rice protoplasmic cells with PIP2-mCherry, a plasma membrane marker and H2B-mCherry, a nuclear marker, respectively. After culturing for 16 h at 28 °C in darkness, fluorescence signals were observed as described in [45].

## Supplementary Information

**Additional file 1: Table S1.** List of the 72 SiSAUR genes identified in this study.

**Additional file 2: Table S2.** Secondary structure prediction and subcellular localization of SiSAURs infoxtail millet.

**Additional file 3: Table S3.** Subfamiliesand protein sequences of Arabidopsis thaliana and Oryza sativa.

**Additional file 4: Table S4.** Proteinmotif analysis of SAURs gene family in foxtail millet.

**Additional file 5: Table S5.** Cis-regulatory elements in the promoter region of SAUR genes.

**Additional file 6: Table S6.** Nine pairs of segmental duplicates in the S. italica SAUR genes.

**Additional file 7: Table S7.** One-to-oneorthologous relationships between Setaria italica and Solanum lycopersicum.

**Additional file 8: Table S8.** The relevant gene primers in this study.

## Data Availability

The entire *Setaria italica* genome sequence information was from the Ensembl Genomes website (http://ensemblgenomes.org/). The *Setaria italica* materials (Yugu 1) used in the experiment were supplied by Li Junxia of the Henan Academy of Agriculture Sciences. The datasets supporting the conclusions of this article are included within the article, figures and additional files.

## Abbreviations

SAUR: Small auxin-up RNA
DST: Downstream element
BLAST: Basic local alignment search tool
aa: Amino acids
KDa: Kilo Dalton
pI: Protein isoelectric point
ABA: Abscisic acid
IAA: Auxin
GA: Gibberellin
MeJA: Methyl jasmonate
SA: Salicylic acid
ABRE: Abscisic acid responsiveness element
LRT: Low temperature response
MBS: MYB binding site
RY-element: Seed specific regulation
TC-rich repeats: Stress responsiveness
O_2_-site: Zein metabolism regulation elements
LG: Linkage groups
qRT-PCR: Quantitative Real-time Polymerase Chain Reaction
